# Preparation of an Immunoaffinity Column Based on Bispecific Monoclonal Antibody for Aflatoxin B_1_ and Ochratoxin A Detection Combined with ic-ELISA

**DOI:** 10.3390/foods11030335

**Published:** 2022-01-25

**Authors:** Disha Lu, Xu Wang, Ruijue Su, Yongjian Cheng, Hong Wang, Lin Luo, Zhili Xiao

**Affiliations:** Guangdong Provincial Key Laboratory of Food Quality and Safety, College of Food Science, South China Agricultural University, Guangzhou 510642, China; louteksa@163.com (D.L.); 18574851733@163.com (X.W.); YSQ20210721@163.com (R.S.); 18815593698@163.com (Y.C.); gzwhongd@163.com (H.W.); lin.luo@scau.edu.cn (L.L.)

**Keywords:** aflatoxin B_1_, ochratoxin A, bispecific monoclonal antibody, immunoaffinity column, ic-ELISA

## Abstract

A novel and efficient immunoaffinity column (IAC) based on bispecific monoclonal antibody (BsMAb) recognizing aflatoxin B_1_ (AFB_1_) and ochratoxin A (OTA) was prepared and applied in simultaneous extraction of AFB_1_ and OTA from food samples and detection of AFB_1_/OTA combined with ic-ELISA (indirect competitive ELISA). Two deficient cell lines, hypoxanthine guanine phosphoribosyl-transferase (HGPRT) deficient anti-AFB_1_ hybridoma cell line and thymidine kinase (TK) deficient anti-OTA hybridoma cell line, were fused to generate a hybrid-hybridoma producing BsMAb against AFB_1_ and OTA. The subtype of the BsMAb was IgG_1_ via mouse antibody isotyping kit test. The purity and molecular weight of BsMAb were confirmed by SDS-PAGE method. The cross-reaction rate with AFB_2_ was 37%, with AFG_1_ 15%, with AFM_1_ 48%, with AFM_2_ 10%, and with OTB 36%. Negligible cross-reaction was observed with other tested compounds. The affinity constant (Ka) was determined by ELISA. The Ka (AFB_1_) and Ka (OTA) was 2.43 × 10^8^ L/mol and 1.57 × 10^8^ L/mol, respectively. Then the anti-AFB_1_/OTA BsMAb was coupled with CNBr-Sepharose, and an AFB_1_/OTA IAC was prepared. The coupling time and elution conditions of IAC were optimized. The coupling time was 1 h with 90% coupling rate, the eluent was methanol–water (60:40, *v*:*v*, pH 2.3) containing 1 mol/L NaCl, and the eluent volume was 4 mL. The column capacities of AFB_1_ and OTA were 165.0 ng and 171.3 ng, respectively. After seven times of repeated use, the preservation rates of column capacity for AFB_1_ and OTA were 69.3% and 68.0%, respectively. The ic-ELISA for AFB_1_ and OTA were applied combined with IAC. The IC_50_ (50% inhibiting concentration) of AFB_1_ was 0.027 ng/mL, the limit of detection (LOD) was 0.004 ng/mL (0.032 µg/kg), and the linear range was 0.006 ng/mL~0.119 ng/mL. The IC_50_ of OTA was 0.878 ng/mL, the LOD was 0.126 ng/mL (1.008 µg/kg), and the linear range was 0.259 ng/mL~6.178 ng/mL. Under optimum conditions, corn and wheat samples were pretreated with AFB_1_-OTA IAC. The recovery rates of AFB_1_ and OTA were 95.4%~105.0% with ic-ELISA, and the correlations between the detection results and LC-MS were above 0.9. The developed IAC combined with ic-ELISA is reliable and could be applied to the detection of AFB_1_ and OTA in grains.

## 1. Introduction

Mycotoxins are natural secondary metabolites produced by filamentous fungi under suitable conditions, among which aflatoxin B_1_ (AFB_1_) and ochratoxin A (OTA) are the most toxic and exist widely in grains [1,2,3,4]. In 2012, aflatoxins were listed in the group 1 classification (carcinogenic to humans) by the International Agency for Research on Cancer (IARC), and OTA was classified as group 2B (possibly carcinogenic to humans) by IARC in 1993 [5]. Some research has indicated that AFB_1_ and OTA frequently and simultaneously contaminated the same grain and the toxic synergistic effect caused aggravated harm [6,7,8].

Considering the health impact of AFB_1_ and OTA, the European Union (EU) has set the maximum level of AFB_1_ at 5.0 µg/kg in maize or rice, and 2.0 µg/kg in other cereals. As for OTA, the maximum level is 3.0~5.0 µg/kg in cereals [9]. In China, the maximum level for AFB_1_ is mandated at 5.0~20 µg/kg in different cereals, and for OTA this is 5.0 µg/kg in all cereals [10]. Detection technology for mycotoxins in food is needed to detect and monitor mycotoxins effectively. Due to the advantages of low cost, easy operation and speed, immunoassay has become the ideal choice in large-scale screening of mycotoxins.

The preparation of antibodies in an immunoassay for mycotoxin is critical [11], and all are reported as single-specific, including polyclonal antibody [12,13], monoclonal antibody (MAb) [14,15,16,17,18] and recombinant antibody [19,20,21,22]. Compared with a single-specific antibody which can only recognize a single analyte in a complex food matrix, a bispecific monoclonal antibody (BsMAb) with two intrinsic specific binding sites could simultaneously recognize and bind two distinct antigens [23,24,25]. It is predominant in immunoassays for food safety detection [25,26,27], which could reduce the cost of combined use of single-specific antibodies.

Several BsMAbs have been reported and applied to establish quantitative or qualitative immunoassays in the food safety field. Wang et al. [28] developed a BsMAb-based multi-analyte enzyme-linked immunosorbent assay (ELISA) for 5-morpholinomethyl-3-amino-2-oxazolidone (AMOZ), malachite green (MG), and leuco-malachite green (LMG) detection in aquatic products. Jin et al. [29] reported a visual colloidal gold immunochromatographic strip with BsMAb to detect carbofuran and triazophos.

In addition with regards to establishing an immunoassay method, BsMAb also has significant application value in other fields of immunoassay. Heterogeneous matrix effects in food affect the detection result and lead to the reduction of sensitivity and accuracy [30,31]. This is especially the case in trace quantity analysis of mycotoxins in grains. An immunoaffinity column (IAC) could separate and concentrate mycotoxins in grain and effectively improve the matrix effect based on a specific and reversible interaction between antigen and antibody [32,33,34]. This may be one of the most applicable and adaptable procedures for mycotoxin detection [35]. IACs have been reported as having extracted mycotoxins in various matrices [36], such as cereals [21,34,37,38], spices [39], potables [40,41], and nuts [42]. The IACs available at present are all based on a single-specific antibody, which can only conjugate to one target compound. Simultaneous contamination of multiple mycotoxins often exists in many foods. In order to extract these mycotoxins, more IACs are needed for sample pretreatment, which would be complicated, time-consuming, and costly. Two or more different antibodies were conjugated in the same IAC in some studies [43,44], which could also recognize and extract two or more mycotoxins simultaneously. Nevertheless, more than one antibody was still necessary, and the preparation or the assay procedures were complex. Moreover, the coupling ratio of different antibodies is uncertain and hard to measure, and mutual interference may be caused by competitive binding of different antibodies. In comparison with single-specific antibody-based IAC, a BsMAb based IAC would be more efficient, convenient, and economical when the sample is contaminated with two mycotoxins. Moreover, the coupling efficiency of BsMAb based IAC is easy to measure, and the interference caused by using two or more different antibodies could be effectively avoided because of the homogeneity of the antibody structure. At present, no BsMAb based IAC has been reported. In this study, in order to further investigate the preparation and properties of BsMAb, and explore its application prospects in IAC, a BsMAb against AFB_1_ and OTA was generated and characterized. Based on this BsMAb, an IAC was prepared and applied in simultaneous extraction of AFB_1_ and OTA from food samples. The extraction conditions were optimized. Then the IAC was applied combined with ic-ELISA (indirect competitive ELISA) for AFB_1_ and OTA detection in food samples.

## 2. Materials and Methods

### 2.1. Reagents and Material

Aflatoxin B_1_ (AFB_1_), ochratoxin A (OTA), aflatoxin B_2_ (AFB_2_), aflatoxin G_1_ (AFG_1_), aflatoxin G_2_ (AFG_2_), aflatoxin M_1_ (AFM_1_), aflatoxin M_2_ (AFM_2_), ochratoxin B (OTB), ochratoxin C (OTC), zearalenone (ZEN), deoxynivalenol (DON), T-2 toxin (T-2) and fumonisin B_1_ (FB_1_), N-methyl-N’-nitro-N-nitrosoguanidine (MNNG), Methyl Methane-sulfonate (MMS), 8-Azaguanine (8-AG), 5-bromo-2′-deoxyuridine (5-BrdU), Paraffin liquid, 50% PEG 4000, goat anti-mouse Immunoglobulin G horseradish peroxidase conjugate (HRP-IgG) were purchased from Sigma-Aldrich Chemical Co. (St. Louis, MO, USA). RPMI Medium 1640 basic (1×), HAT Media Supplement (50×), HT Media Supplement (50×), Pierce Rapid Isotyping Kits-Mouse were purchased from Thermo Fisher Scientific Co., Ltd. (Waltham, MA, USA). Fetal bovine serum was purchased from Guangzhou Saiguo Biotech Co., Ltd. (Guangzhou, China). All other chemicals and solvents were of analytical grade and were purchased from Shanghai Aladdin Bio-Chem Technology Co., Ltd. (Shanghai, China). Water was prepared using a Milli-Q water purification system.

CNBr-activated Sepharose 4B and polyethylene columns (8.9 mm × 63 mm) were purchased from Pharmacia Corporation (Wuhan, China). Female Balb/c mice (10 weeks old) were purchased from Guangdong Medical Laboratory Animal Centre (Foshan, China). Anti-AFB_1_ hybridoma cell line E_4_, anti-OTA hybridoma cell line B_3_, anti-AFB_1_ MAb (IgG_1_), anti-OTA MAb (IgG_1_), AFB_1_-OVA, OTA-OVA, blocking buffer, P solution and TMB chromogenic solution were produced by our laboratory.

### 2.2. Apparatus

ELISA plates were washed using a Multiskan MK2 microplate washer (Thermo Fisher Scientific Inc., Waltham, MA, USA). Absorbances of ELISA were measured on a Multiskan MK3 microplate reader (Thermo Fisher Scientific Inc., Waltham, MA, USA). Absorbances of antibody concentrations were measured on a NanoDrop 2000 c Ultraviolet spectrometer (Thermo Fisher Scientific Inc., Waltham, MA, USA).The LC-MS assay was conducted using an Agilent 6400 series LC system and an ECLIPS PLUS C_18_ (2.1 × 100 mm, 1.8 µm) (Agilent Technologies, Santa Clara, CA, USA) with a Triple Quadrupole Mass Spectrometer.

### 2.3. Mutagenesis of HGPRT and TK Deficient Hybridoma Cell Lines

The chemical mutagen scheme was performed as follows [28,45,46]: AFB_1_ hybridoma cell line E_4_ was cultured in HAT medium for 2 d before mutagenic treatment in a humidified 5% CO_2_ incubator at 37 °C. The cells were washed twice with PBS and cultured in HT medium for 2 d to restore the cells to normal morphology and were cultured in HAT medium for 24 h. After washing the cells with PBS twice, the cells were mutagenized in different concentrations of MNNG (5.0 μg/mL, 10.0 μg/mL, 20.0 μg/mL, and 40.0 μg/mL). The mutagenic treatment time was 2 h, 4 h, 6 h and 10 h. At the same time, the control group was cultured in 1‰ DMSO complete medium (RPMI-1640 culture media containing 20% fetal bovine serum) without MNNG. The cells were washed twice with PBS to finish the mutagenesis and cultured in complete medium. Finally, the cells were cultured for 3 d and the survival rate of the cells was determined by trypan blue staining, and the combination of MNNG concentration and mutagenic treatment time resulting in a survival rate of about 70% were selected as the optimal mutation parameters. OTA hybridoma cell line B_3_ was mutated with MMS (1.0 μg/mL, 5.0 μg/mL, 10.0 μg/mL, and 20.0 μg/mL) and for 2 h, 4 h, 6 h and 10 h by the same steps as above.

### 2.4. Screening of HGPRT and TK Deficient Hybridoma Cell Lines

After mutagenic treatment with MNNG, AFB_1_ hybridoma cells E_4_ were collected and incubated in a 6-well culture plate in selective semi-solid media containing 6-TG (50 μg/mL). After 10 d, the visible white clonal cell clusters were placed into a 96-well culture plate and cultured with 200 μL complete media containing 6-TG (50 μg/mL). The cell supernatants were analyzed by ELISA to screen positive clones which could recognize and bound to AFB_1_. Then the cells were tested by HAT media to confirm their HAT sensitivity. If the cell line could not grow in HAT media, it was HGPRT-deficient, and was named E_4_-HGPRT^−^. Via the same steps, the TK-deficient OTA hybridoma cell line named B_3_-TK^−^ was screened with 5-BrdU (30 μg/mL).

### 2.5. Generation and Characterization of BsMAb

#### 2.5.1. Generation of BsMAb

E_4_-HGPRT^−^ and B_3_-TK^−^ were fused under the effect of PEG 4000 [47]. The fused cells were cultured in 96-well culture plates with HAT media for 10 d. The cell supernatants were tested by ic-ELISA to confirm the presence of BsMAb which could recognize and bound to AFB_1_ and OTA simultaneously.

The selected tetra-doma cell lines were cloned by 4 rounds of limiting dilution assays, and then inoculated into female Balb/c mice that had been primed with 500 μL of sterile liquid paraffin. Ascites was collected from the Balb/c mice and purified with Protein G to obtain BsMAb. The concentration of BsMAb was measured by a NanoDrop 2000 c ultraviolet spectrometer. The subtype of BsMAb was determined by Pierce Rapid Isotyping Kits-Mouse. The purity and molecular weight of BsMAb were estimated by the SDS-PAGE method.

#### 2.5.2. Antibody Specificity Determination

The cross-reactivity (CR) could be used as an index to evaluate the specificity of the anti-AFB_1_/OTA BsMAb. Inhibition curves were fitted through ic-ELISA data to determine the IC_50_ (50% inhibiting concentration) of each common mycotoxin contaminant (aflatoxin B_1_, aflatoxin B_2_, aflatoxin G_1_, aflatoxin G_2_, aflatoxin M_1_, aflatoxin M_2_, ochratoxin A, ochratoxin B, ochratoxin C, zearalenone, deoxynivalenol, T-2 toxin and fumonisin B_1_). The CR was calculated using the following equation:

(1) $$\mathrm{CR}=\frac{{\mathrm{IC}}_{50}\;\mathrm{of}\;{\mathrm{AFB}}_{1}\;\mathrm{or}\;\mathrm{OTA}}{{\mathrm{IC}}_{50}\;\mathrm{of}\;\mathrm{common}\;\mathrm{mycotoxin}\;\mathrm{contaminant}}\times 100\%$$

#### 2.5.3. Antibody Affinity Determination

The affinity of BsMAb was validated as follows [48]: the coating antigen AFB_1_-OVA and OTA-OVA diluted to four gradient concentrations (5.0 μg/mL, 2.5 μg/mL, 1.25 μg/mL, 0.625 μg/mL) were coated in microplates at 4 °C for 14 h, respectively. The anti-AFB_1_/OTA BsMAb was diluted to eight gradient concentrations (9.6 × 10^−3^ mg/mL, 4.8 × 10^−3^ mg/mL, 2.4 × 10^−3^ mg/mL, 1.2 × 10^−3^ mg/mL, 6.0 × 10^−4^ mg/mL, 3.0 × 10^−4^ mg/mL, 1.5 × 10^−4^ mg/mL, 7.5 × 10^−5^ mg/mL) with PBS buffer, and then added to the micropores for specific binding. After reacting with HRP-IgG as secondary antibody, chromogenic solution, and termination solution successively, the A_450nm_ was determined. The reaction curve was fitted by Origin 8.5 software. The concentration of BsMAb was used as abscissa and A_450nm_ as ordinate. The average value of BsMAb concentration corresponding to the half-saturation index was taken as the affinity constant (Ka) of BsMAb.

### 2.6. Preparation of BsMAb Based IAC

According to the instruction, the procedures of IAC preparation was as follow: 0.5 g of CNBr-Sepharose 4B powder was swelled with 3 mL of HCl (1 mM) and washed with 20 mL of coupling buffer (0.1 M NaHCO_3_, pH 8.3) 5 times. Then, the Sepharose gel was mixed with 2 mL anti-AFB_1_/OTA BsMAb solution (9.61 mg in coupling buffer) and gently stirred at 25 °C. The concentration of unconjugated BsMAb in supernatant was determined by NanoDrop 2000 c ultraviolet spectrometer every 0.5 h to calculate the coupling efficiency. The optimal coupling time was determined when the coupling efficiency was over 90%.

The coupling efficiency was calculated as follows:

(2) $$\mathrm{Coupling}\;\mathrm{Efficiency}=\frac{\mathrm{Concentration}\;\mathrm{of}\;\mathrm{BsMAb}\;\mathrm{before}\;\mathrm{coupling}-\mathrm{Concentration}\;\mathrm{of}\;\mathrm{unconjugated}\;\mathrm{BsMAb}}{\mathrm{Concentration}\;\mathrm{of}\;\mathrm{BsMAb}\;\mathrm{Before}\;\mathrm{Coupling}}$$

The agarose gel was redissolved in 50 mL blocking buffer (0.1 M Tris-HCl, pH 8.0) to block the free active sites at 25 °C for 2 h, and washed with 20 mL HAc-NaAc buffer (0.1 M, pH 4.0) and 20 mL Tris-HCl buffer (0.1 M Tris-HCl, pH 8.0) alternately for 4 cycles. Finally, 1 mL of immune gel prepared above was transferred to a polyethylene column and balanced with 20 mL loading buffer (0.1 M PBS, pH 7.4). The IAC prepared was stored with PBS containing 0.01% Merthiolate sodium (*v*/*w*) at 4 °C until use.

### 2.7. Optimization of Elution Conditions and Evaluation of the IAC Capacity

Mixed standard solution containing 50 ng AFB_1_ and 50 ng OTA was diluted in 15 mL loading buffer and drawn through the IAC. Then 20 mL of loading buffer was applied to wash the IAC. Finally, elution buffer was used to elute the analyte bound to the column. The collected eluent diluted twice with PBS was detected by ic-ELISA and recovery rates were evaluated.

Three elution conditions were optimized to determine the optimum conditions, including type of elution buffer, concentration of methanol in elution buffer, and volumes of elution buffer. Elution buffer A is 0.2 M glucine solution, pH 3.0. Elution buffer B is methanol-water, 90:10, *v*:*v*. Elution buffer C is methanol-water, 60:40, *v*:*v*, pH 2.3. Elution buffer D is methanol-water, 60:40, *v*:*v*, pH 2.3, containing 1 M NaCl. The concentration of methanol in elution buffer are 30%, 40%, 50%, and 60% (*v*/*v*). The volumes of elution buffer are 1 mL, 2 mL, 3 mL, 4 mL, 5 mL.

Under the optimal conditions, 15 mL of loading buffer containing excessive mixed standard of AFB_1_ (300 ng) and OTA (300 ng) was passed through the IAC. The eluent diluted with PBS was detected by ic-ELISA. The column was used repeatedly for several cycles, and the column capacity and preservation rates were calculated and compared. The column capacity/preservation rate was expressed as follows:

(3) $$\mathrm{Column}\;\mathrm{Capacity}=\frac{\mathrm{Concentration}\;\mathrm{of}\;{\mathrm{AFB}}_{1}\;\mathrm{or}\;\mathrm{OTA}\times \mathrm{Loading}\;\mathrm{Amount}}{\mathrm{Immune}\;\mathrm{Gel}\;\mathrm{Volume}}$$

(4) $$\mathrm{Preservation}\;\mathrm{Rate}=\frac{\mathrm{Column}\;\mathrm{Capacity}\;\mathrm{for}\;\mathrm{Several}\;\mathrm{Cycles}}{\mathrm{Column}\;\mathrm{Capacity}\;\mathrm{for}\;\mathrm{the}\;\mathrm{First}\;\mathrm{Treatment}}$$

### 2.8. ic-ELISA Combined with IAC

**Figure 1:**
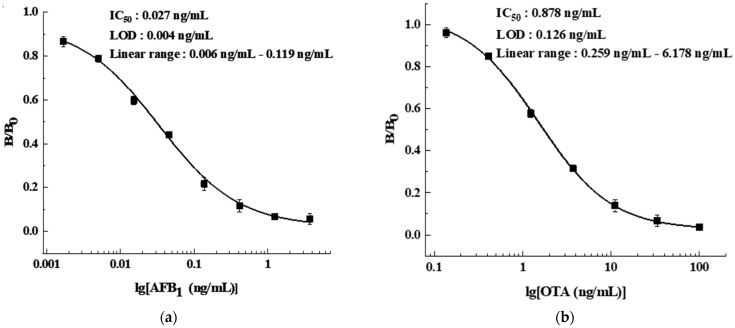
The inhibition curve of ic-ELISA: (**a**) AFB_1_; (**b**) OTA.

The collected eluent was detected by the ic-ELISA developed and optimized in our laboratory. The parameters of ic-ELISA are shown in Table 1 and the procedures are as follows. Coating antigen solution was coated in a 96-well polystyrene microtiter plate with 100 μL/well at 4 °C for 14 h, then washed with 300 μL/well wash solution (PBS containing 0.05% Tween-20) twice. 120 μL/well blocking buffer was used to block uncoated sites at 37 °C for 3 h, and then the plate was dried in a draught drying cabinet at 37 °C. Then, 50 μL MAb solution and 50 μL AFB_1_/OTA standard solution of gradient concentrations or the collected eluent diluted twice with PBS were added to each well and incubated, and the plate was washed 4 times. 100 μL/well HPR-IgG was added for incubation, and then washed 4 times. After washing, 100 μL/well of TMB chromogenic solution was added into each well and incubated for 10 min at 37 °C, and 50 μL/well of 2 M H_2_SO_4_ was added to terminate the chromogenic reaction. The absorbance at 450 nm (A_450nm_) was measured. The A_450nm_ of the well without standard solution was as B_0_ and with standard solution was as B. The inhibition curve was fitted by Origin 8.5 software adopting B/B_0_ versus the concentration of AFB_1_ or OTA, and the IC_50_ was estimated (Figure 1). The limit of detection (LOD) was defined as the IC_10_ value. The linear range was taken as IC_20_ to IC_80_. The IC_50_ of AFB_1_ was 0.027 ng/mL, the LOD was 0.004 ng/mL (0.032 µg/kg), and the linear range was 0.006 ng/mL~0.119 ng/mL. The IC_50_ of OTA was 0.878 ng/mL, the LOD was 0.126 ng/mL (1.008 µg/kg), and the linear range was 0.259 ng/mL~6.178 ng/mL. The LODs of the ic-ELISA to AFB_1_ and OTA were obviously lower than the maximum levels. Therefore, this method was suitable for quantitative detection of AFB_1_ and OTA.

### 2.9. Analysis of Corn and Wheat Samples

The pretreatment method for AFB_1_ and OTA analysis was carried out as follows [43]. Corn and wheat samples were evenly crushed and passed through a 40-mesh sieve. 5.0 g of sample was put into a 50 mL polypropylene centrifuge tube, and 25 mL of methanol-water (70:30, *v*:*v*, containing 2.4% NaCl) was added, and shaken periodically for 5 min. After centrifuging at 4000 rpm for 10 min, the supernatant was collected through a 0.45 μm PTEE membrane filter to obtain the sample extract. Then 5 mL of the sample extract was diluted with 10 mL loading buffer and drawn through the AFB_1_/OTA-IAC. Nonspecifically adsorbed impurities were washed off using 20 mL loading buffer. Then, elution buffer optimized in 2.7 was used to elute, and diluted twice with PBS before detection by ic-ELISA. Concurrently, the samples were detected with LC-MS.

LC-MS conditions were modified from published methods [49] as follows: an Agilent LC system coupled with an Agilent 6400 series triple Quadrupole mass spectrometer was used for the confirmatory analysis. An Agilent ECLIPS PLUS C_18_ (2.1 × 100 mm, 1.8 µm) with solvent consisted of 0.1% formic acid (mobile phase A) and acetonitrile (mobile phase B, 0–2 min, 30%; 2–5 min, 90%; 5–10 min, 5%) at a flow rate of 0.30 mL/min. The temperature was 40 °C and the injection volume was 5 μL. The analysis was performed using positive-ion electrospray interface (ESI) with a multiple reaction monitoring (MRM) mode. The retention times of AFB_1_ and OTA were 3.339 min and 3.94 min, respectively.

Blank corn and wheat samples without AFB_1_ and OTA, having been confirmed by LC-MS, were used to spike recovery experiments. AFB_1_ and OTA of different concentrations (4 μg/kg, 10 μg/kg, 20 μg/kg, 50 μg/kg, 100 μg/kg) were spiked into the blank samples. Eight grain samples were randomly selected from a local market, including four corn samples and four wheat samples. After pretreatment, the sample eluents were detected by ic-ELISA, and the results were compared with those of LC-MS.

## 3. Results

### 3.1. Mutagenesis and Screening of HGPRT and TK Deficient Hybridoma Cell Lines

**Figure 2:**
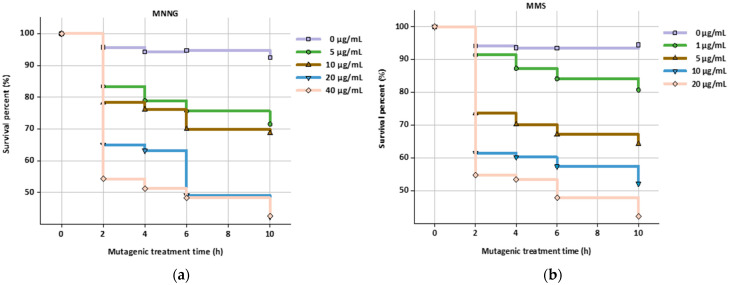
Effects of mutagen concentration and mutagenic treatment time on survival percentage (%) of the hybridoma cell line. (**a**) AFB_1_ hybridoma cell line E_4_; (**b**) OTA hybridoma cell line B_3_.

The AFB_1_ hybridoma cell line E_4_ and OTA hybridoma cell line B_3_ were applied for construction of a HGPRT and TK deficient mutant, respectively. When the concentration of MNNG was 10 μg/mL and the treatment time was 6 h, the cell survival percentage was 69.9% (Figure 2a), which was determined as the optimal mutation condition of E_4_ cells. When the concentration of MMS was 5 μg/mL and the treatment time was 4 h, the cell survival percentage was 70.2% (Figure 2b), and the optimal mutation condition for B_3_ cells was determined. As mutagens, MNNG and MMS have strong mutagenic effect on cells. Over-high concentration and overlong treating time will reduce the activity of cells, or even result in apoptosis.

**Figure 3:**
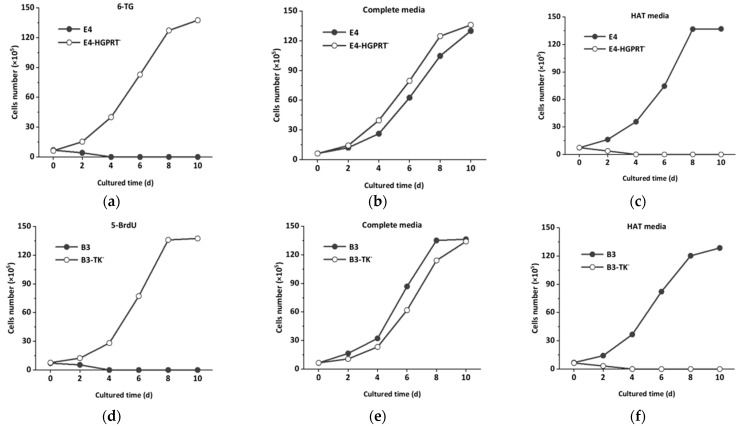
The growth curve of AFB_1_ hybridoma cell lines E_4_ and OTA hybridoma cell lines B_3:_ (**a**) cell line E_4_ and E_4_-HGPRT^−^ in 6-TG; (**b**) cell line E_4_ and E_4_-HGPRT^−^ in the complete media; (**c**) cell line E_4_ and E_4_-HGPRT^−^ in HAT media; (**d**) cell line B_3_ and B_3_-TK^−^ in 5-BrdU; (**e**) cell line B_3_ and B_3_-TK^−^ in the complete media; (**f**) cell line B_3_ and B_3_-TK^−^ in HAT media.

After MNNG mutation treatment, the E_4_ cells were inoculated in 6-TG medium for selective screening. The screened E_4_-HGPRT^−^ cell line gradually formed colonies with the ability to fission and grow. Finally, stable inherited deficient cells E_4_-HGPRT^−^ were obtained. Similarly, stable inherited deficient cells B_3_-TK^−^ were screened in 5-BrdU. Figure 3 depict the growth curves of E_4_-HGPRT^−^ and B_3_-TK^−^ in screening reagent (6-TG and 5-BrdU), complete media and HAT media after mutagenic treatment. The unmutated cells could not survive in screening reagent (Figure 3a,d). In complete medium culture, both normal cell line and mutant could grow normally (Figure 3b,e). The cell apoptosis in HAT medium was adopted as an indication of HGPRT or TK deficiency. The growth curve (Figure 3c,f) shows that E_4_-HGPRT^−^ and B_3_-TK^−^ were unable to divide in HAT media, which indicates that E_4_-HGPRT^−^ and B_3_-TK^−^ were sensitive to HAT media.

### 3.2. Generation and Characterization of BsMAb

#### 3.2.1. Generation of BsMAb

**Figure 4:**
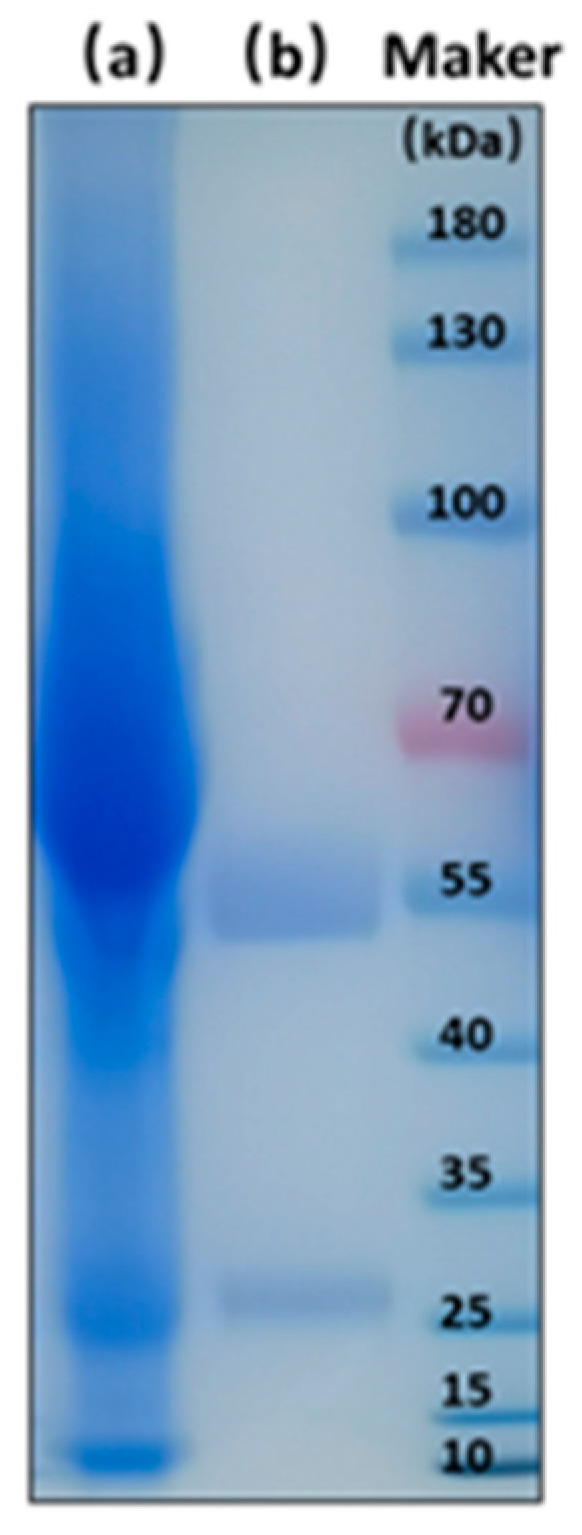
The electropherogram of BsMAb: (**a**) ascites; (**b**) purified BsMAb.

E_4_-HGPRT^−^ and B_3_-TK^−^ were fused by hybrid-hybridoma technology. A total of 83 tetradoma colonies were generated, and the cell supernatant was identified by ELISA. After 3–4 rounds of subcloning, a positive tetradoma T26 was chosen and was used for production of anti-AFB_1_/OTA BsMAb by induction in vivo. Clear bands of heavy chain (about 55 kDa) and light chain (about 25 kDa) from BsMAb were observed in the electropherogram (Figure 4). The concentration of BsMAb was 9.61 mg/mL measured by NanoDrop ultraviolet spectrophotometer.

#### 3.2.2. Antibody Specificity Determination

The cross-reactivity of the BsMAb with other common mycotoxins was detected by ELISA. As shown in Table 2, five mycotoxins containing similar structures displayed evident cross-reactivities. The cross-reaction rate with AFB_2_ was 37%, with AFG_1_ 15%, with AFM_1_ 48%, with AFM_2_ 10%, and with OTB 36%. Negligible cross-reaction was observed with other tested compounds.

It may be speculated from the cross-reactivities of aflatoxins that the specific recognition site of the BsMAb to aflatoxins was mainly coumarin plus cyclopentenone, followed by the difuran ring. The double bond on the furan ring was more conducive to the recognition of the antibody than the single bond (the cross-reaction rate of AFG_1_ was higher than that of AFG_2_, and the same trend exists between AFM_1_ and AFM_2_). Very often anti-AFB_1_ antibodies have high cross-reactivity for other aflatoxins, and a positive result for AFB_1_ is a sufficient motive for detailed analysis of aflatoxins in the sample by other techniques [43]. IAC was applied as pretreatment method in this study, and a certain amount of cross-reactivity of the antibody would be more acceptable than that used in specific quantitative analysis.

#### 3.2.3. Antibody Affinity Determination

**Figure 5:**
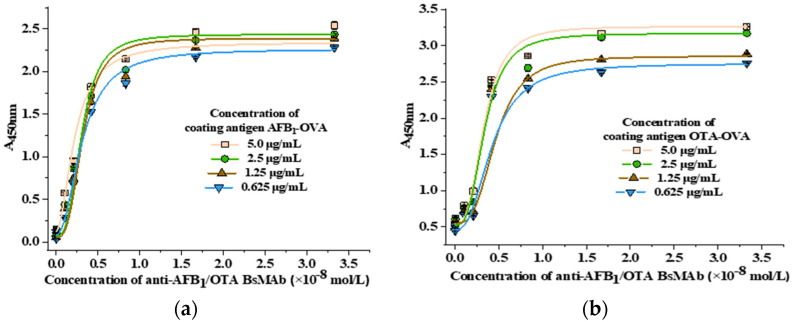
The affinity constants (Ka) of anti-AFB_1_/OTA BsMAb: (**a**) AFB_1_; (**b**) OTA.

The affinity constant (Ka) at different coating concentrations was confirmed by ELISA and shown in Figure 5. The average Ka of the BsMAb to AFB_1_ and OTA were 2.43 × 10^8^ L/mol, and 1.57 × 10^8^ L/mol, respectively. Previous studies indicated that the affinity constant (Ka) of high affinity antibodies is between 10^7^ L/mol–10^12^ L/mol [48], suggesting that the BsMAb prepared in this study is a high affinity antibody.

### 3.3. Preparation of BsMAb Based IAC

**Figure 6:**
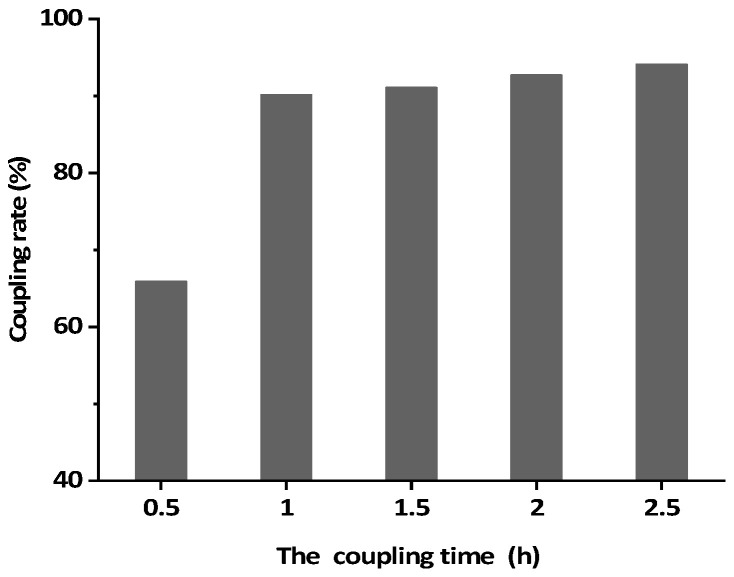
The coupling rate of different coupling times.

While anti-AFB_1_/OTA BsMAb was coupling with CNBr-Sepharose 4B, the concentration of antibody in the supernatant of the coupling solution was determined every 0.5 h, and the coupling efficiency was calculated to determine the optimal coupling time. Figure 6 shows that the coupling rate rapidly increased to 90.1% for 1 h and the increase in speed over 1 h became slower. Thus 1 h was selected as the optimal coupling time.

### 3.4. Optimization of Elution Conditions and Evaluating of the IAC Capacity

To achieve high extraction efficiency, three affecting conditions were optimized and were evaluated by the recovery of AFB_1_ and OTA.

**Figure 7:**
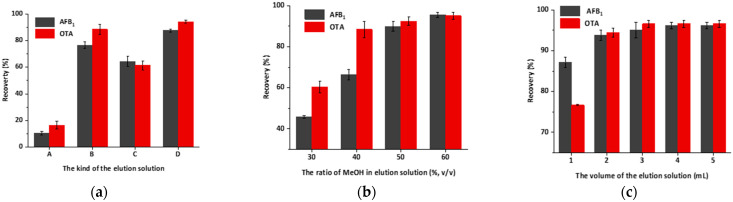
Optimization results of the IAC elution conditions: (**a**) the recovery of AFB_1_ and OTA with different kind of elution solution (*n* = 3); (**b**) the recovery of AFB_1_ and OTA with different ratios of methanol–water as elution solution (*n* = 3); (**c**) The recovery of AFB_1_ and OTA with different elution volume (*n* = 3).

Firstly, four kinds of elution buffer (A, B, C, D) were tested. The recoveries are shown in Figure 7a. Solution A achieved the lowest recovery, containing no methanol and ionic compound. When the column was eluted with solution D, the recovery of AFB_1_ and OTA was up to 90%. The concentration of methanol, pH and ion concentration could all influence the recovery. The methanol concentration has significant effect, and ion concentration and low pH also improved the elution recovery. It can be seen from Figure 7a that the recoveries of solution B and D are all around 80%. However, high methanol concentration may affect the activity of the antibody [43], so solution D containing NaCl and medium concentration of methanol is preferred.

Secondly, to further investigate the effect of methanol concentration, elution solutions containing different concentrations of methanol (30%, 40%, 50% and 60%, *v*/*v*, 1 M NaCl, pH 2.3) were evaluated. The recovery rose along with the increase of methanol concentration, and the highest recovery was obtained when the concentration of methanol was 60% (Figure 7b).

Thirdly, under the conditions optimized above, using solution containing 60% methanol, 1 M NaCl, pH 2.3 as elution solution, the effect of elution solution volumes (1, 2, 3, 4, 5 mL) on recoveries were also investigated. As shown in Figure 7c, recoveries increased from 87.2% to 96.2% for AFB_1_ and 76.7% to 96.6% for OTA as the volume increased from 1 mL to 4 mL. The highest recovery was acquired when the volume was above 4 mL, and further increase of the volume did not visibly improve the recovery, thus 4 mL was selected as the optimized volume.

Under the optimized elution conditions (4 mL of solution D with 60% methanol and 1 M NaCl used as elution buffer), the IAC was used repeatedly for seven cycles and the capacity was detected at every cycle. As shown in Table 3, the maximum binding capacities of the IAC for AFB_1_ and OTA were both over 165 ng. As the column is used repeatedly, the capacity decreases gradually. After seven cycles of use, the preservation rates of column capacity for AFB_1_ and OTA were 69.3% and 68.0%, respectively (Table 3), which is equivalent to 114.4 μg/kg and 116.3 μg/kg of AFB_1_ and OTA in samples, respectively, by conversion. According to some investigation results of mycotoxin contamination in agro--products and food samples [50,51,52,53], the capacity of the IAC could meet the needs of practical applications.

### 3.5. Analysis of Corn and Wheat Samples

AFB_1_ and OTA in spiked corn and wheat samples were detected by IAC combined with ELISA, and confirmed by LC-MS. The recoveries of IAC-ELISA ranged from 95.4% to 105.0%, and the coefficients of variation (CV) were less than 10% (Table 4). The results indicated that the assay performed well with appropriate recovery and accuracy. This may be ascribed to IAC pretreatment, which could relieve matrix interference effectively and ameliorate the recovery and accuracy of ELISA for AFB_1_ and OTA detection in food matrices. The results were confirmed by LC-MS to further prove the reliability of this assay.

**Figure 8:**
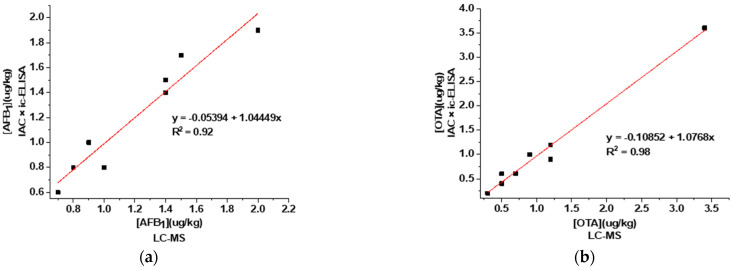
Results of real sample detection by IAC-ELISA and LC-MS: (**a**) AFB_1_; (**b**) OTA.

Eight real samples of corn and wheat were simultaneously analyzed by the established IAC-ELISA and LC-MS. The results are shown in Figure 8. AFB_1_ and OTA both tested positive in the eight samples, but the levels were all below the maximum levels. The correlations between the detection results and LC-MS were both above 0.9. Thus it can be seen that the established IAC-ELISA could be applied to the detection of AFB_1_ and OTA simultaneously in real corn and wheat samples with high accuracy.

## 4. Conclusions

In this study, we develop a BsMAb based IAC which could bind AFB_1_ and OTA simultaneously, and the binding sites were 1:1 with the two mycotoxins. The coupling rate and the binding site ratio of AFB_1_ and OTA are easy to adjust and measure, and mutual interference could be avoided because of the homogeneity of the antibody structure. It is more efficient, convenient, and economical than the IACs based on single-specific antibodies. With satisfactory matrix effect elimination effect and recovery rate, it could be applied to detect AFB_1_ and OTA rapidly and effectively, combined with ic-ELISA. Besides, the application of this anti-AFB_1_/OTA BsMAb in IAC could provide reference for the application of other BsMAb in IAC in the future.

The BsMAb prepared in this study demonstrated different cross-reactivities to five biotoxins with similar structures. It is necessary to further improve the hapten structures and the screening strategy to prepare a more specific BsMAb if it were to be applied in the field with higher requirements for specificity. As a mature ELISA detection mode that could quantitatively determine two analytes simultaneously is unavailable at present, the ic-ELISA used in this study can just detect AFB_1_ and OTA respectively. If simultaneous detection of two toxins in ELISA can be achieved, this study will have more practical application value.

## Figures and Tables

**Table 1:** Parameters for ic-ELISA.

Target Analyte	AFB_1_	OTA
Coating antigen	AFB_1_-OVA	OTA-OVA
Coating concentration (μg/mL)	0.31	0.26
Coating buffer	0.05 M carbonate buffer (pH 9.6)	
Coating condition	14 h, 4 °C	
MAb	Anti-AFB_1_ MAb	Anti-OTA MAb
Standard analyte	AFB_1_	OTA
Competition condition	30 min, 37 °C	
HRP-IgG dilution	1:5000	
HRP-IgG incubation condition	30 min, 37 °C	

**Table 2:** IC_50_ and cross-reactivity (CR) of anti-AFB_1_/OTA BsMAb against related mycotoxins.

Mycotoxin Analyte	Structure	IC_50_ (ng/mL)	CR (%)
AFB_1_	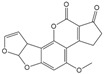	0.037	100
AFB_2_	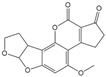	0.101	37
AFG_1_	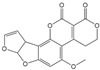	0.252	15
AFG_2_	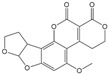	3.584	1
AFM_1_	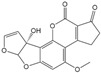	0.077	48
AFM_2_	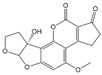	0.377	10
OTA	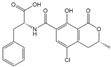	2.040	100
OTB	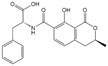	5.620	36
OTC	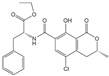	201.535	1
ZEN	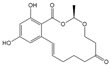	>1000	<0.1
DON	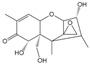	>1000	<0.1
FB_1_	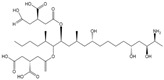	>1000	<0.1
T-2	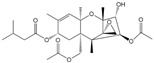	>1000	<0.1

**Table 3:** Column capacity and preservation rate of AFB_1_ and OTA in 7 cycles (*n* = 3).

Cycle	AFB_1_		OTA	
	Column Capacity (ng)	Preservation Rate (%)	Column Capacity (ng)	Preservation Rate (%)
1	165.0	100.0	171.1	100.0
2	162.5	98.5	169.5	99.1
3	159.0	96.4	166.4	97.3
4	153.6	93.1	156.2	91.3
5	147.5	89.4	145.8	85.2
6	128.9	78.1	133.3	77.9
7	114.4	69.3	116.3	68.0

**Table 4:** Results of spiked recovery experiments of corn and wheat samples with IAC-ELISA and LC-MS (*n* = 2).

Samples	Analyte	Spiked Concentration (μg/kg)	IAC-ELISA			LC-MS		
			Measured (μg/kg)	Recovery (%)	CV (%)	Measured (μg/kg)	Recovery (%)	CV (%)
Corn 0	AFB_1_	0	ND ^a^	NC ^b^	NC	ND	NC	NC
	OTA	0	ND	NC	NC	ND	NC	NC
Corn 1	AFB_1_	100	95.6	95.6	5.0	97.3	97.3	3.3
	OTA	100	98.6	98.6	4.5	97.9	97.9	3.5
Corn 2	AFB_1_	50	49.2	98.4	4.7	49.8	99.6	3.3
	OTA	50	49.1	98.2	4.4	49.4	98.8	3.1
Corn 3	AFB_1_	20	19.2	96.0	4.3	19.2	96.0	3.5
	OTA	20	19.6	98.0	4.4	19.2	96.0	3.2
Corn 4	AFB_1_	10	9.9	99.0	3.4	9.8	98.0	3.8
	OTA	10	10.3	103.0	4.2	9.8	98.0	3.6
Corn 5	AFB_1_	4	4.1	102.5	2.6	3.8	95.0	3.2
	OTA	4	4.2	105.0	2.4	3.9	97.5	3.5
Wheat 0	AFB_1_	0	ND	NC	NC	ND	NC	NC
	OTA	0	ND	NC	NC	ND	NC	NC
Wheat 1	AFB_1_	100	99.1	99.1	4.8	98.2	98.2	3.1
	OTA	100	98.9	98.9	4.6	97.5	97.5	3.1
Wheat 2	AFB_1_	50	47.7	95.4	4.5	48.7	97.4	3.8
	OTA	50	47.7	95.4	3.3	49.2	98.4	3.5
Wheat 3	AFB_1_	20	19.5	97.5	4.0	19.2	96.0	3.2
	OTA	20	19.4	97.0	3.3	19.7	98.5	3.1
Wheat 4	AFB_1_	10	9.6	96.0	3.3	9.8	98.0	3.8
	OTA	10	9.9	99.0	3.2	9.9	99.0	3.5
Wheat 5	AFB_1_	4	3.9	97.5	3.1	3.9	97.5	3.2
	OTA	4	3.9	97.5	2.8	4.0	100.0	3.8

^a^ ND, not detectable. ^b^ NC, not calculated.

## Data Availability

Data is contained within the article.
